# Transcriptional and Post-Transcriptional Regulation of Proangiogenic Factors by the Unfolded Protein Response

**DOI:** 10.1371/journal.pone.0012521

**Published:** 2010-09-02

**Authors:** Ethel R. Pereira, Nan Liao, Geoff A. Neale, Linda M. Hendershot

**Affiliations:** 1 Department of Genetics & Tumor Cell Biology, St. Jude Children's Research Hospital, Memphis, Tennessee, United States of America; 2 Department of Molecular Sciences, University of Tennessee Health Science Center, Memphis, Tennessee, United States of America; 3 Hartwell Center for Bioinformatics and Biotechnology, St. Jude Children's Research Hospital, Memphis, Tennessee, United States of America; University of Hong Kong, Hong Kong

## Abstract

**Background:**

Inadequate extracellular conditions can adversely affect the environment of the ER and impinge on the maturation of nascent proteins. The resultant accumulation of unfolded proteins activates a signal transduction pathway, known as the unfolded protein response, which serves primarily to protect the cell during stress and helps restore homeostasis to the ER.

**Principal Findings:**

Microarray analysis of the unfolded protein response in a human medulloblastoma cell line treated with thapsigargin revealed that, in addition to known targets, a large number of proangiogenic factors were up-regulated. Real-Time PCR analyses confirmed that four of these factors, VEGFA, FGF2, angiogenin and IL8, were transcriptionally up-regulated in multiple cell lines by various ER stress inducers. Our studies on *VEGFA* regulation revealed that XBP-1(S), a UPR-inducible transcription factor, bound to two regions on the *VEGFA* promoter, and analysis of XBP-1 null mouse embryonic fibroblasts revealed that it contributes to *VEGFA* expression in response to ER stress. ATF4, another UPR-inducible transcription factor, also binds to the *VEGFA* gene, although its contribution to *VEGFA* transcription appeared to be fairly modest. We also found that *VEGFA* mRNA stability is increased in response to UPR activation, via activation of AMP kinase, demonstrating that increased mRNA levels occur at two regulatory points. In keeping with the mRNA levels, we found that VEGFA protein is secreted at levels as high as or higher than that achieved in response to hypoxia.

**Conclusions and Significance:**

Our results indicate that the UPR plays a significant role in inducing positive regulators of angiogenesis. It also regulates *VEGFA* expression at transcriptional, post-transcriptional and post-translational levels and is likely to have widespread implications for promoting angiogenesis in response to normal physiological cues as well as in pathological conditions like cancer.

## Introduction

Changes in the extracellular environment of a cell can adversely affect the normal homeostasis of the endoplasmic reticulum (ER), which disrupts the folding and processing of secretory pathway proteins. The resulting accumulation of unfolded proteins in the ER increases the demands for molecular chaperones and folding enzymes and activates a signal transduction cascade known as the unfolded protein response (UPR) [1]. This multi-component signal transduction pathway is largely cytoprotective; serving to decrease the detrimental effects of accumulated unfolded proteins by increasing molecular chaperones that bind to them, decreasing protein synthesis to limit the accumulation, and finally increasing the degradative capacity of the cell to eliminate them. However if normal homeostasis is not restored during prolonged stress conditions, the UPR can induce apoptosis in these cells in order to protect the organism [1], [2]. In mammalian cells, the UPR is controlled by three resident ER transmembrane proteins that “sense” ER stress and activate signals to downstream elements; Ire-1, PERK and ATF6. Ire-1 is an ER localized transmembrane protein, which has a kinase and endoribonuclease domain in its cytosolic tail. On sensing ER stress, Ire-1 is phosphorylated in *trans*, which in turn activates its endonuclease domain leading to the excision of 26 bases from the X-box binding protein (XBP-1) transcript [3]. The resulting frame shift encodes a fully active transcription factor XBP-1(S), which up-regulates expression of a number of resident ER proteins that contribute to folding or degradation of unfolded or misfolded proteins [4], [5]. In addition to Ire-1 signaling, mammalian cells also transiently inhibit cap-dependent protein translation and arrest cells in the G1 phase of the cell cycle through activation of the PKR-like ER kinase (PERK) [6], [7]. Contrary to this global inhibition in protein translation occurring in PERK-activated cells, synthesis of the ATF4 transcription factor is increased during ER stress [6]. ATF4 transactivates expression of a number of genes including CHOP [8], a pro-apoptotic protein, and GADD34 [9], which reverses the block in translation. PERK also activates NFκB [10], a pro-survival protein, thus contributing to the balance between survival and death signals. Lastly, activation of ATF6 results in its translocation to the Golgi and cleavage by the S1P and S2P proteases to release the cytosolically oriented active transcription factor that up-regulates expression of XBP-1, as well as folding enzymes and ER chaperones, such as PDI and BiP [11], [12].

In addition to protecting cells during physiological and chemical conditions that adversely affect protein folding in the ER, there is increasing evidence to show that the UPR also plays an important role in normal development and physiology. This includes liver development [13], plasma cell differentiation [14], [15], bone development [16], [17], and normal pancreatic homeostasis [18]. Mice that are null for either XBP-1 [13] or its upstream activator Ire1α [19], [20], [21], [22] die at day E12.5 due to hepatoinsufficiency. In both cases, this was later confirmed to be due to an inability to produce XBP-1(S), a major regulator of hepatic development. In addition to liver, pancreas, and muscle, XBP-1(S) is also highly expressed in the placenta [19], and Ire1α null embryos show evidence of placental abnormalities. To determine the role of Ire1 in this tissue, a recent study generated mice lacking Ire1α by crossing Ire1α^+/−^ mice with *Mox2^+/Cre^* transgenic mice [19]. Mox2 is ubiquitously expressed except in the labyrinthine trophoblasts of the placenta. This allowed Ire1-deficient embryos to be produced that have normal levels of Ire1 in the placenta [19]. This study revealed that loss of Ire1α in the placenta led to decreased vascular endothelial growth factor (VEGFA) production, which is a major inducer of angiogenesis, thereby resulting in severe dysfunction of this highly vascularized tissue.

Angiogenesis refers to the sprouting, migration and remodeling of existing blood vessels [23] and plays an important role in a number of normal physiological processes including embryonic development, wound healing, and the female reproductive cycle. It also plays a role in several pathological conditions including ischemia and cancer. Angiogenesis is regulated by a fine balance between factors that stimulate the formation of new blood vessels and those that inhibit it [24], [25]. Proangiogenic factors such as VEGF, fibroblast growth factors (FGFs), platelet derived growth factors (PDGFs), and IL8 are released by cancer cells experiencing decreased oxygen and nutrient supplies [26], [27], [28]. These factors act as ligands that bind to specific receptors on endothelial cells, causing them to proliferate and to release matrix metalloproteinases that degrade the extracellular matrix, allowing them to migrate toward the angiogenic stimulus in order to establish new blood vessels [26]. The predominant and best studied proangiogenic factor is VEGFA, a homodimeric heparin binding glycoprotein that is produced in several isoforms due to alternative splicing. The different isoforms of VEGFA (206, 189, 165, 145 and 121) have varying expression patterns and contrasting properties [29]. Of these VEGF_165_ is the predominant and best characterized isoform, and plays an important role in mediating angiogenesis [30]. All VEGF isoforms are synthesized and processed in the endoplasmic reticulum (ER) and transported through the secretory pathway [26], [31].

The HIF (hypoxia inducible factor) pathway is the best characterized cellular stress pathway that leads to the up-regulation of proangiogenic factors in response to inadequate oxygen delivery [32]. HIF-1 and HIF-2 are heterodimeric transcription factors consisting of an oxygen-labile α subunit and a constitutively expressed β subunit. Hypoxia stabilizes the α subunit, thereby activating the HIF complex, which in turn binds to the promoters of target genes such as *VEGF* and other proangiogenic factors and transactivates them [33]. Prolonged hypoxia can also increase *VEGFA* mRNA stability leading to further increases in VEGFA production [34]. In addition to the role of the HIF signaling pathway in up-regulating *VEGF* expression, several recent studies demonstrate that the UPR also contributes to *VEGF* transcription [35] and protein processing in the ER [36]. Using microarray analysis, we found that in addition to *VEGFA* a large number of proangiogenic factors were up-regulated by UPR inducers. The up-regulation of several of these factors by ER stress was as robust as, or even greater than, that achieved with hypoxia. We found that two UPR-regulated transcription factors bound directly to the *VEGFA* promoter in response to ER stress and contributed to its transcription. In addition, activation of AMP kinase stabilized the *VEGFA* transcripts, further contributing to *VEGFA* mRNA levels. Our finding that a number of regulators of angiogenesis are a target of the UPR argues that this physiological process should be added to the growing list of normal homeostatic and developmental processes that this stress pathway controls.

## Results

### UPR activation results in the transcriptional up-regulation of a number of proangiogenic factors

We wished to characterize the UPR in a solid tumor cell line that could ultimately be used in xenograft studies to ensure that this stress response was fully active and that all branches were intact. To do so, we treated Daoy, a human medulloblastoma cell line with thapsigargin, a Ca^2+^ ATPase inhibitor and potent inducer of the UPR, and performed genome-wide microarray analyses. Overall, we identified 1069 probe sets with differential expression after either 3 or 8 hours of thapsigargin treatment compared to untreated cells. Further analysis of this data confirmed significant enrichment of the expected UPR target genes, including ER chaperones, folding enzymes, and proteins involved in ER associated degradation (ERAD), as well as the transcription factors that are known to up-regulate them in response to ER stress. In addition to UPR targets, somewhat unexpectedly, gene ontology analysis revealed a significant enrichment of genes associated with the regulation of angiogenesis. A total of 185 genes on the array are annotated as being associated with angiogenesis. As many of these encode endothelial cell-specific proteins or cell surface receptors on endothelial cells, we limited our further analysis to the 33 genes that are secreted proteins or transcription factors that either positively or negatively regulate angiogenesis. Of the 19 genes that are characterized as positive regulators of angiogenesis, 13 showed a greater than 2-fold increase in expression in at least one time point after thapsigargin-treatment (Table 1). Additionally expression of one negative regulator of angiogenesis, vasohibin *(VASH1)* was decreased with ER stress. These data suggest that regulating angiogenesis is likely to be a major function of the UPR.

**Table 1:** UPR activation enhances expression of proangiogenic factors.

			Fold change Tg treated	
Gene Symbol	Gene Name	Angiogenic Effect	3 hour	8 hour
ANG	Angiogenin	Positive	2.4	8.1
ANGPT2	Angiopoietin-2	Positive	−2.6	2.2
CTGF	Connective tissue growth factor	Positive	2.2	1.4
EPAS1	Endothelial Pas domain protein 1 (HIF2α)	Positive	1.8	2.6
EREG	Proepiregulin	Positive	2.3	6.3
FGF2	Fibroblast growth factor-2	Positive	1.5	3.1
F3	Thromboplastin	Positive	2.9	1.6
IL1A	Interleukin-1 α	Positive	4.4	10.8
IL6	Interleukin-6	Positive	4.8	7
IL8	Interleukin-8	Positive	54.25	27.9
KLF5	Kruppel-like factor 5	Positive	2.6	3.5
TGFB2	Transforming growth factor beta-2	Positive	4.1	2.9
VEGFA	Vascular endothelial growth factor A	Positive	1.7	2.7
VASH1	Vasohibin	Negative	−1.3	−3

### Comparison of UPR inducers with hypoxia in the up-regulation of proangiogenic factors

**Figure 1:**
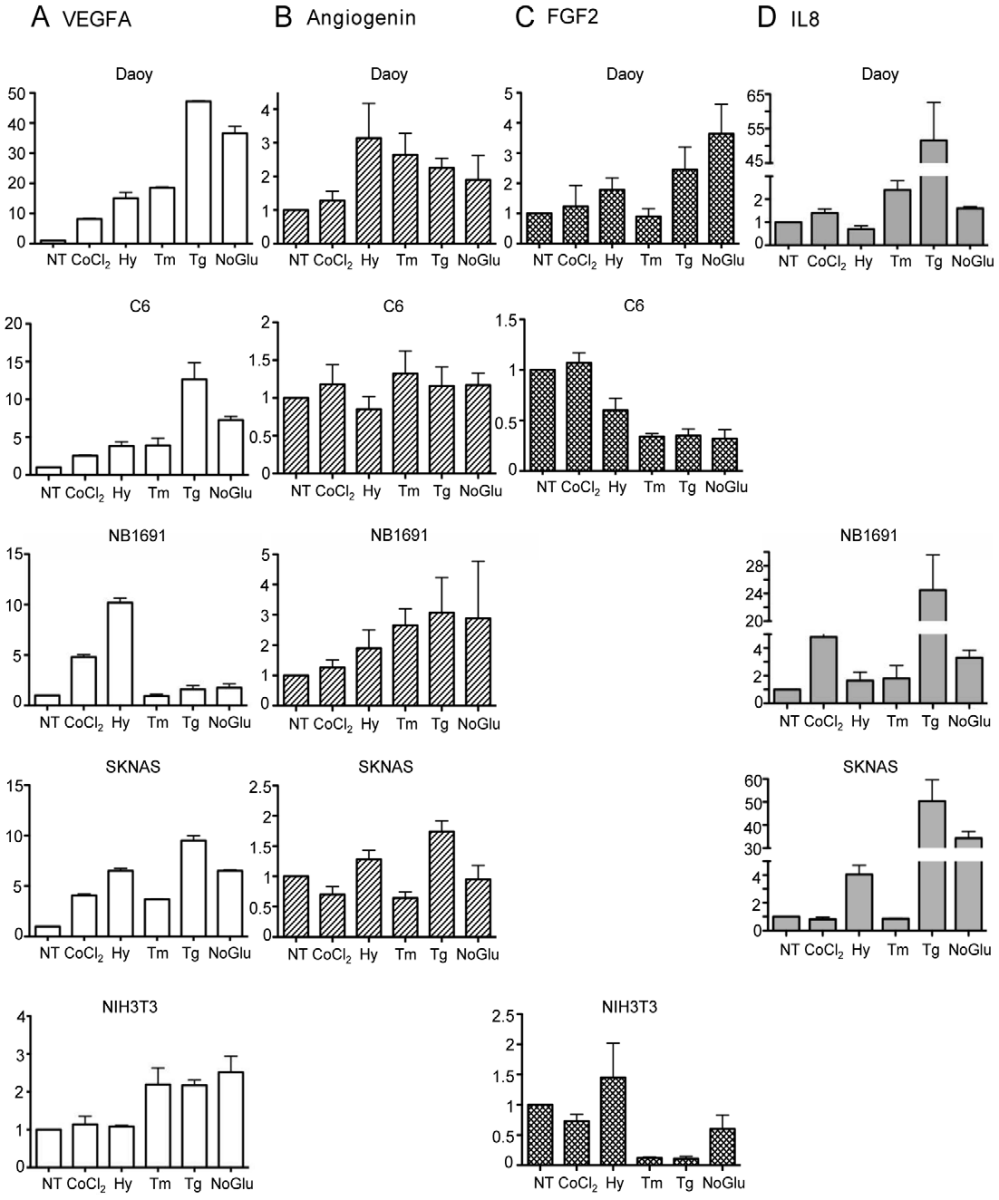
Up-regulation of proangiogenic factor mRNA by the UPR and hypoxia. Daoy, NB1691, SKNAS, C6 and NIH3T3 cells were treated with 100 µM CoCl_2_, 1% O_2_ hypoxia (Hy), 2.5 µg/ml tunicamycin (Tm), 1 µM thapsigargin (Tg), or no glucose media (No Glu) for 24 hours. RNA was extracted for qRT-PCR analysis and mRNA fold induction relative to the untreated control sample, which was set to 1, was determined for (**A**) VEGF (white bars) (**B**) angiogenin (striped bars) (**C**) FGF2 (chequered bars) and (**D**) IL8 (grey bars). Experiments were performed in triplicate (values are mean ± SD).

To confirm the induction of proangiogenic factors by the UPR, we treated cell lines with UPR inducers and compared the magnitude of their induction to that achieved with conditions that activate the HIF pathway using quantitative Real-Time PCR (qRT-PCR) (Figure 1). We confirmed by western blot analyses that CoCl_2_ and the level of hypoxia (1% O_2_) used in our experiments induced HIF1α and BNIP3, its downstream target, but did not induce UPR targets. Importantly, the UPR inducing agents did not activate the HIF signaling pathway (Figure S1). Thus the conditions we used in our analysis allowed us to specifically activate these two stress pathways independently. Five different cell lines were treated with a variety of UPR inducers (*e.g*., tunicamycin, thapsigargin, and no glucose) and with two different inducers of the HIF pathway *(e.g.,* CoCl_2_ and 1% oxygen) for 24 hours, and the induction of four of the best characterized proangiogenic factors: VEGFA, bFGF, angiogenin and IL8 was measured. We also confirmed that downstream UPR target genes like *CHOP* and *BiP* mRNA were up-regulated by ER stress in each of the cell lines tested (Figure S2). As expected, all four factors were up-regulated by hypoxic conditions, although the magnitude varied dramatically between cell lines, largely due to differences in their basal levels of synthesis (Figure S3). When UPR inducers were used, we found that in many cases the induction of the four proangiogenic factors was nearly as high as or even higher than that achieved with hypoxia, although there were some interesting differences. Hypoxia was a strong inducer of *VEGFA* mRNA in the NB1691 neuroblastoma cell line, while ER stress had little effect on *VEGFA* levels. Conversely ER stress induced *VEGFA* in the NIH3T3 fibroblast line, but hypoxia did not (Figure 1A). Similarly ER stress induced *FGF2* expression greater than hypoxia in the Daoy line, whereas neither stress condition stimulated its production in the C6 and NIH3T3 cell lines, perhaps due to the high levels of basal expression of *FGF2* in these two lines (Figure 1C). In keeping with the microarray data, angiogenin was modestly induced in the Daoy cell line and the NB1691 line, but in the other three lines there was very little effect with either hypoxia or ER stress inducers (Figure 1B), again in keeping with higher basal levels in these lines. Finally, increases in human-specific *IL8* expression were much more dramatic with ER stress than with hypoxia in all three human cell lines (Figure 1D).

### UPR activation increases *VEGFA* mRNA stability via AMPK

**Figure 2:**
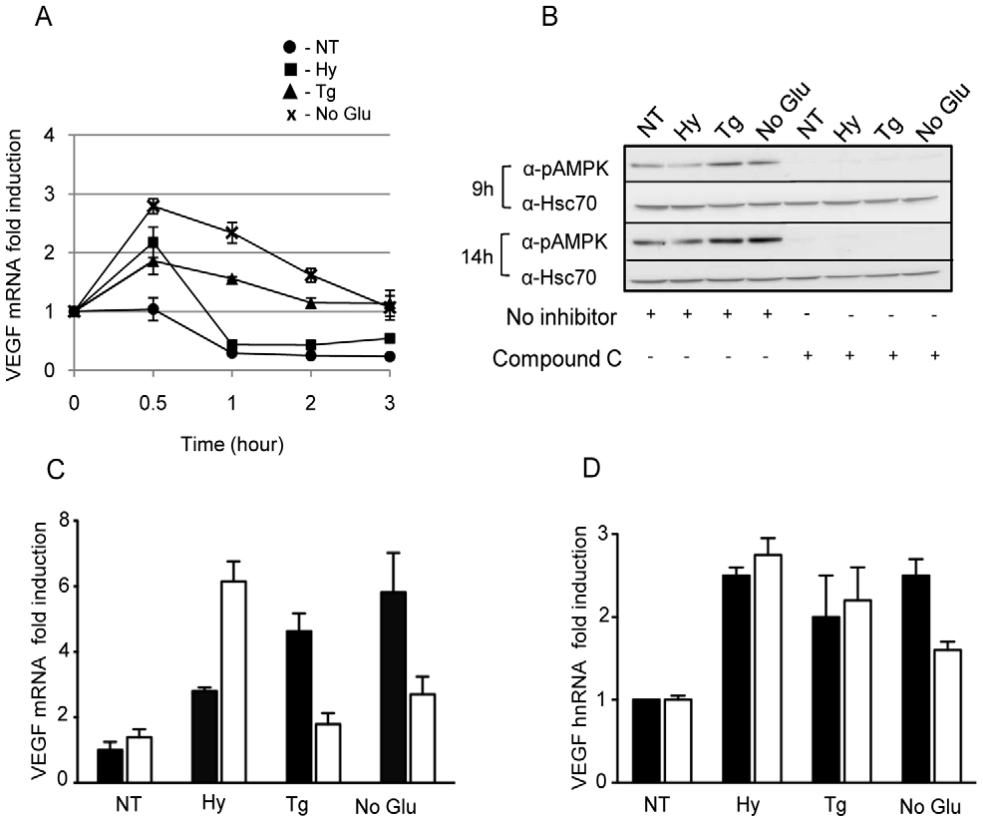
UPR activation stabilizes *VEGF* mRNA via AMPK. (**A**) C6 cells were pre-treated with normal culture conditions (NT-circle), 1% O_2_ (Hy-square), thapsigargin (Tg-triangle), or no glucose media (No Glu-cross) for 6 hours. Actinomycin D (5 µg/ml) was added to the various cultures to block further transcription. At the indicated times, total RNA was extracted and subjected to qRT-PCR to determine *VEGF* mRNA levels. The mean values of data from duplicate experiments are presented (± SD). (**B**) C6 cells were either left untreated or treated as indicated in the figure in the presence or absence of Compound C for 9 hours or 14 hours. Western blot analysis was performed on cell lysates to determine levels of p-AMPK. Hsc70 was used as loading control. (**C–D**) C6 cells were pretreated with different stress inducers for 6 hours as indicated. No inhibitor (black) or compound C (AMPK inhibitor- white) was added to the cells as indicated for an additional 8 hours. Total RNA was extracted for qRT-PCR to determine *VEGF* mRNA (**C**) and *VEGF* hnRNA (**D**) levels. Experiments were performed in triplicate (values are mean ±SD).

In this study we focused on determining the mechanism by which the UPR regulates *VEGFA* expression, as VEGFA is the best characterized stimulator of angiogenesis and represents a therapeutic target for treating cancer as well as several ischemic, infectious and inflammatory disorders [37]. Additionally, we favored this target because in most of the lines we examined, including mouse cells, *VEGFA* was induced to higher levels with ER stress than with hypoxia. We chose the C6 cell line for these experiments, because it had a low basal expression of VEGF which was potently induced by ER stress, previous studies used this line to study VEGF gene regulation by hypoxia, and this line was used in xenograft studies to determine the role of ORP150/GRP170 in VEGF processing and secretion. *VEGFA* mRNA levels increase in response to hypoxic conditions via a combination of an enhanced transcription iaat early time points coupled with an increase in the stability of the mRNA at later times [38]. To investigate whether UPR activation might also increase *VEGFA* mRNA stability, we examined the turnover of *VEGFA* mRNA under control and various stress conditions (Figure 2A). Cells were pretreated with hypoxia or two different UPR inducers and then incubated with actinomycin D to inhibit further transcription. In control cells the low level of basal transcripts were rapidly degraded in keeping with previous studies [34], [39]. For all three stress inducers, there was a reproducible increase in *VEGFA* mRNA at 30 minutes after adding actinomycin D, which is compatible with an increase in transcription occurring before the inhibitor takes effect. We found that although hypoxic conditions led to an initial increase in VEGFA levels, the mRNA was rapidly degraded. This is consistent with a previous report using C6 cells, which showed that hypoxia had no significant effect on the half-life of *VEGFA* mRNA until much later time points [38]. When cells were pretreated with the two UPR inducers, we found that after the initial burst in *VEGFA* transcripts they decayed significantly slower than in control or hypoxia-treated cells. (Figure 2A), arguing that ER stress leads to increased *VEGFA* mRNA stability at relatively early times in the response.

The increase in *VEGFA* mRNA stability observed after prolonged exposure to hypoxic conditions is due to the binding of a hypoxia-inducible protein complex, such as HuR, to the ARE (adenylate-uridylate rich elements) region in the 3'UTR region of *VEGFA* mRNA [34]. Additionally, stress activated protein kinases such as AMPK, p38MAPK, JNK, and PI3K have been implicated in increasing *VEGFA* mRNA stability through their action on the AU rich region of the 3'UTR [40], [41], [42], [43]. We used a variety of kinase inhibitors to determine if any of their targets might play a role in increasing the stability of *VEGFA* mRNA during ER stress. When the UPR-activated cells were incubated with compound C, an AMP kinase inhibitor, there was a significant reduction in *VEGFA* transcripts (Figure 2C), suggesting that this kinase played a role in the UPR-induced stabilization of *VEGFA*. Activation of AMPK by ER stress was confirmed by western blotting, as was the efficacy of its inhibitor, Compound C (Figure 2B). We also co-incubated UPR activated cells with inhibitors of the PI3 and JUN kinases, but found that they had no affect on *VEGFA* mRNA levels in response to UPR activation (data not shown). As an additional control, the effect of the AMPK inhibitor on *VEGFA* mRNA levels was examined in cells pre-treated with hypoxia for 6 h (Figure 2C), which was previously shown to be not long enough to stabilize *VEGFA* transcripts [38]. Unexpectedly, we found that *VEGFA* stability was actually increased in hypoxia treated cells that we incubated with Compound C, although we do not understand the basis for this effect. To verify that the effects of this inhibitor was specifically on *VEGFA* mRNA stability and did not alter transcription of the *VEGFA* gene, we treated cells with the various combinations of kinase inhibitor and stress inducers and examined heteronuclear *VEGFA* RNA (hnRNA) levels (Figure 2D), which can be used as a measure of transcription. We found that there was no indication that this inhibitor affected *VEGFA* transcription, thus confirming that *VEGFA* transcripts are stabilized during ER stress, which apparently is due to the activation of the AMP kinase.

### UPR activation increases the transcription of *VEGFA*

**Figure 3:**
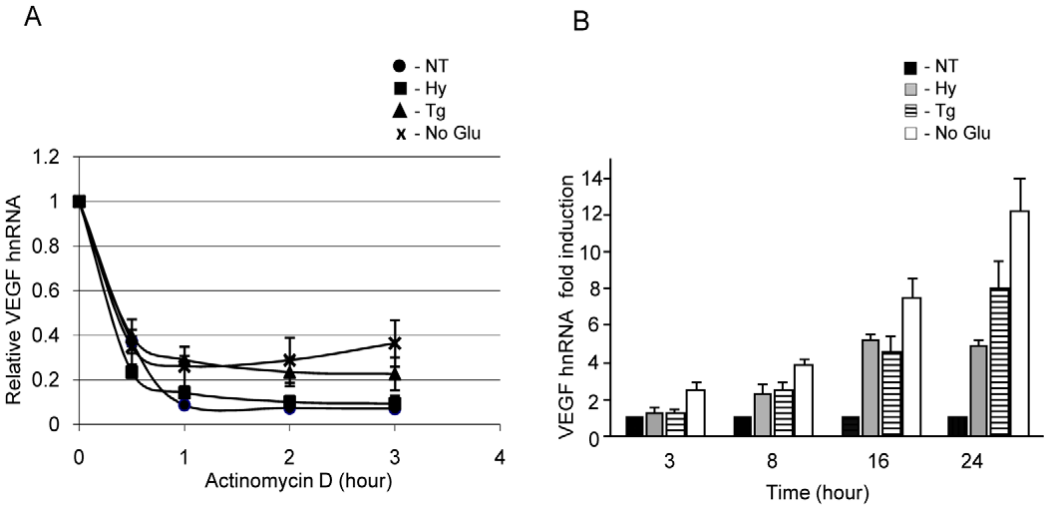
UPR activation increases *VEGF* transcription rate. (**A**) C6 cells were pre-treated with normal culture conditions (NT-circle), 1% O_2_ (Hy-square), thapsigargin (Tg-triangle), or no glucose media (No Glu-cross) for 6 hours as indicated. Actinomycin D (5 µg/ml) was added to block further transcription. At the indicated times, total RNA was extracted and subjected to qRT-PCR to determine the kinetics of the disappearance of *VEGF* hnRNA in control and stressed cells. (**B**) C6 cells were untreated (NT-black), treated with 1% O_2_ (Hy-grey), thapsigargin (Tg-striped) or no glucose media (No Glu-white) for the indicated times. Total RNA was extracted, and VEGF hnRNA was quantitated by qRT-PCR. Experiments were performed in triplicate (values are mean ±SD).

Our analysis of *VEGFA* hnRNA in the experiment described above revealed that the unprocessed hnRNA levels were higher in cells treated with thapsigargin or no glucose than in control cells. This suggested that *VEGFA* might also be transcriptionally regulated in response to ER stress. Prior to using hnRNA levels as a measure of the transcription rate, we first confirmed that the splicing of *VEGFA* mRNA was not significantly affected by UPR activation. Cells were pretreated for 6 hours with the indicated stressors, and actinomycin D was added to inhibit further transcription. Heteronuclear RNA was then measured at the indicated time points. We found that *VEGFA* hnRNA decreased at a fairly similar rate in control and stress activated cells through at least eight hour of treatment, arguing that these stresses did not dramatically affect splicing up to this point (Figure 3A). Therefore, the measurement of *VEGFA* hnRNA could be used as an indication of the transcription rate of this gene in response to UPR activation (Figure 3B). Cells incubated in media containing no glucose, increased *VEGFA* transcription to a much greater extent than either thapsigargin or hypoxia at all time points measured, which is in keeping with the 30 minute time point in Figure 2A. Thapsigargin was as good as or better than hypoxia at inducing *VEGFA* transcription throughout the course of the experiment. Thus, the increased transcription rate, coupled with the stabilization of *VEGFA* transcripts, accounts for the higher steady state level of *VEGFA* mRNA in response to thapsigargin compared to hypoxia in this cell line (Figure 1A). Although the transcription rate of *VEGFA* appeared to be the highest in the presence of no glucose, this is not reflected in the steady state levels after 24 hours of treatment (Figure 1A), which may be due to some inhibition of splicing occurring at later time points (Figure 2A).

### XBP-1 binds to two regions in the rat *VEGFA* promoter

**Figure 4:**
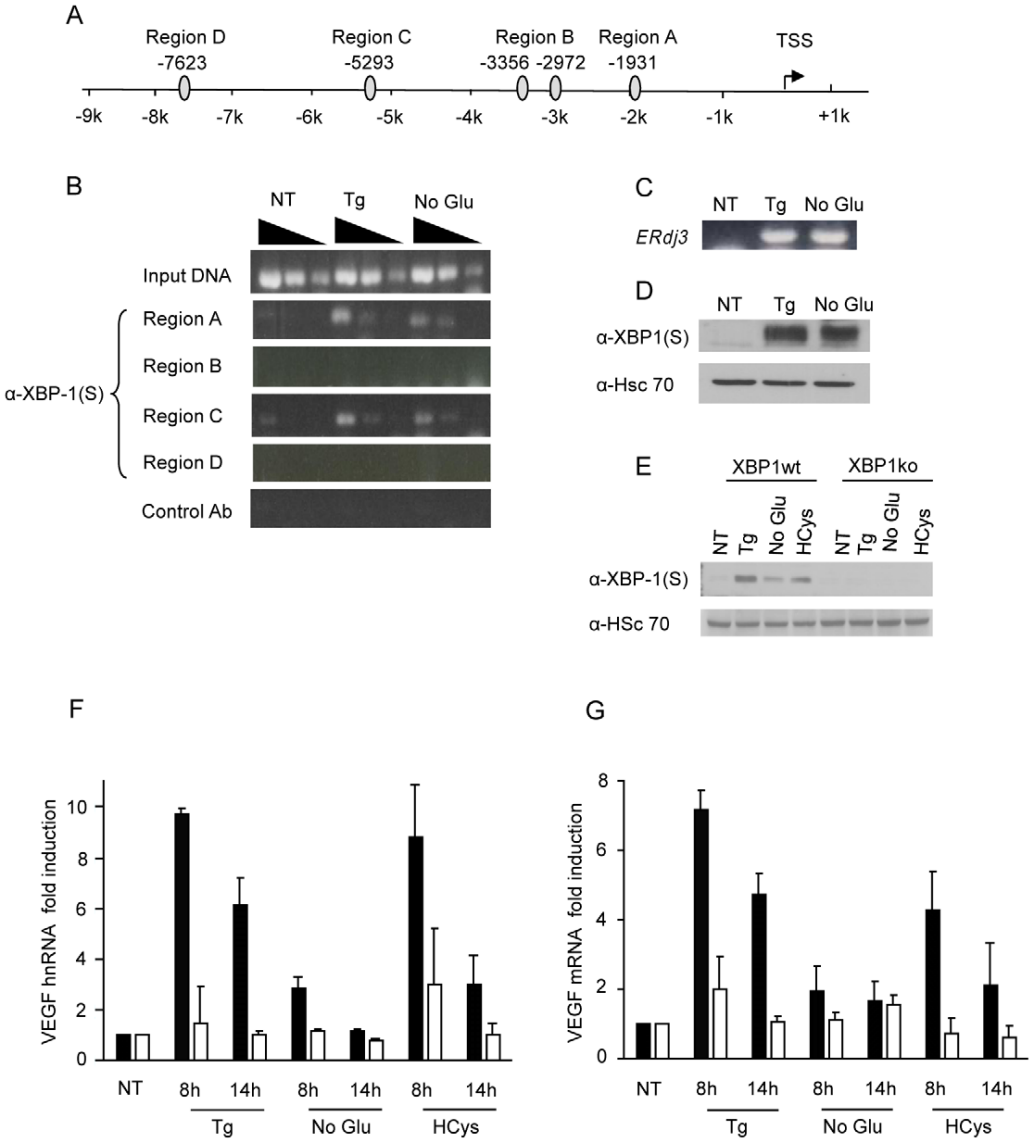
XBP-1 binds to two regions in the rat *VEGF* promoter in response to ER stress and contributes to increasing *VEGF* transcription rate. (**A**) Potential XBP-1 sites in the rat *VEGF* promoter. (**B**) Cross-linked chromatin from C6 cells that were untreated (NT), thapsigargin-treated (Tg), or incubated in no glucose media (No Glu) for 8 hours were immunoprecipitated with anti-XBP-1 or with a control antiserum (anti-BiP). Ten-fold serial dilutions of precipitated chromatin and input controls were used for PCR amplification. (**C**) As a positive control, primers spanning the XBP-1 binding region on the ERdj3 promoter were used to PCR amplify the anti-XBP-1 precipitated chromatin (**D**) XBP-1(S) protein levels were detected in C6 cells treated with Tg and No Glu media using Western blot analysis. (**E**) XBP-1 wild-type (black) or null (white) MEFs were untreated (NT), thapsigargin-treated (1 µM), treated with media lacking glucose (No Glu) or homocysteine-treated (10 mM) for 14 hours. Cell lysates were prepared and XBP-1(S) was detected by western blot analysis. (**F-G**) Cells were treated as in (**E**) and total RNA from the indicated samples was subjected to qRT-PCR to quantify *VEGF* hnRNA (**F**) or *VEGF* mRNA (**G**) at the indicated time points. RNA levels were expressed relative to the control untreated samples for each line, which was set to 1. Experiments were performed in triplicate (values are mean ± SD).

To identify potential binding sites for various UPR-inducible transcription factors, we analyzed the human, mouse and rat *VEGFA* promoters using the computer programs rVista and TRANSFAC (Figure S4). In addition to HIF sites, the promoters of all three species have a number of potential binding sites for the UPR-induced transcription factors XBP-1 and ATF4, whereas only the mouse promoter has a single ATF6 site. We first assessed whether XBP-1(S) bound to any of the five potential sites in the rat promoter in response to ER stress using a chromatin immunoprecipitation (ChIP) assay, since the C6 rat glioma was used for both the mRNA stability and transcription assays. Indeed, XBP-1(S) could be detected at two different sites (i.e., one at ∼1.9 kb and one at ∼5.2 kb up-stream of the transcription start site) in response to both thapsigargin and no glucose treatment (Figure 4B). We were unable to detect XBP-1(S) binding to the remaining three potential sites in these cells upon UPR activation, suggesting that either they are not used or that the anti-XBP-1(S) antiserum used to immunoprecipitate the chromatin could not gain access to these sites. As a positive control, we showed that XBP-1 binds to the ERdj3 promoter (Figure 4C), as documented previously [44]. We detected XBP-1(S) protein in ER stressed but not in untreated C6 cell lysates that were used for the chromatin immunoprecipitation assays (Figure 4D).

### XBP-1 mediates increased expression of *VEGFA* following ER stress

To determine the contribution of XBP-1 to the up-regulation of *VEGFA* transcription, we made use of XBP-1 wild-type (XBP-1 wt) and null (XBP-1 ko) mouse embryonic fibroblasts (MEFs). Examination of these cells by western blotting confirmed that no XBP-1(S) protein could be detected in the XBP-1 null cells in response to UPR induction (Figure 4E). Next we compared the fold induction of both *VEGFA* hnRNA (Figure 4F) and *VEGFA* mRNA (Figure 4G) in both cell lines after treating with three different ER stress inducers; thapsigargin, no glucose media, and homocysteine. Our qRT-PCR analysis in the XBP-1 wild-type MEFs demonstrated an ∼10 fold increase in *VEGFA* transcription rate after 8 h of either thapsigargin or homocysteine treatment (Figure 4F); whereas no glucose media was a relatively poor inducer of *VEGFA* transcription in this cell line, perhaps in keeping with the reduced amount of XBP-1(S) produced by this stress condition (Figure 4E). The transcription rate was highest at 8 h for both thapsigargin and homocysteine treatment, demonstrating that its induction is not sustained during UPR activation in the wild-type MEFs as was observed in the C6 cell line. This is mirrored in the total mRNA transcripts, which were also higher at 8 hrs of stress induction (Figure 4G). When the XBP-1 null cells were similarly examined, we found that there was little or no increase in *VEGFA* transcription with any of the treatments, suggesting that XBP-1 played a major role in the up-regulation of *VEGFA* in response to ER stress. However, closer analysis of the *VEGFA* hnRNA data from the two cell lines revealed that the untreated XBP-1 wild-type MEFs had a lower basal level of *VEGFA* hnRNA than the null cells, (Figure S5). Hence, the exact contribution of XBP-1 in up-regulating *VEGFA* was somewhat complicated by the differences in basal transcription rates between the two lines.

### ATF4 contributes to up-regulation of *VEGFA* expression following UPR activation

**Figure 5:**
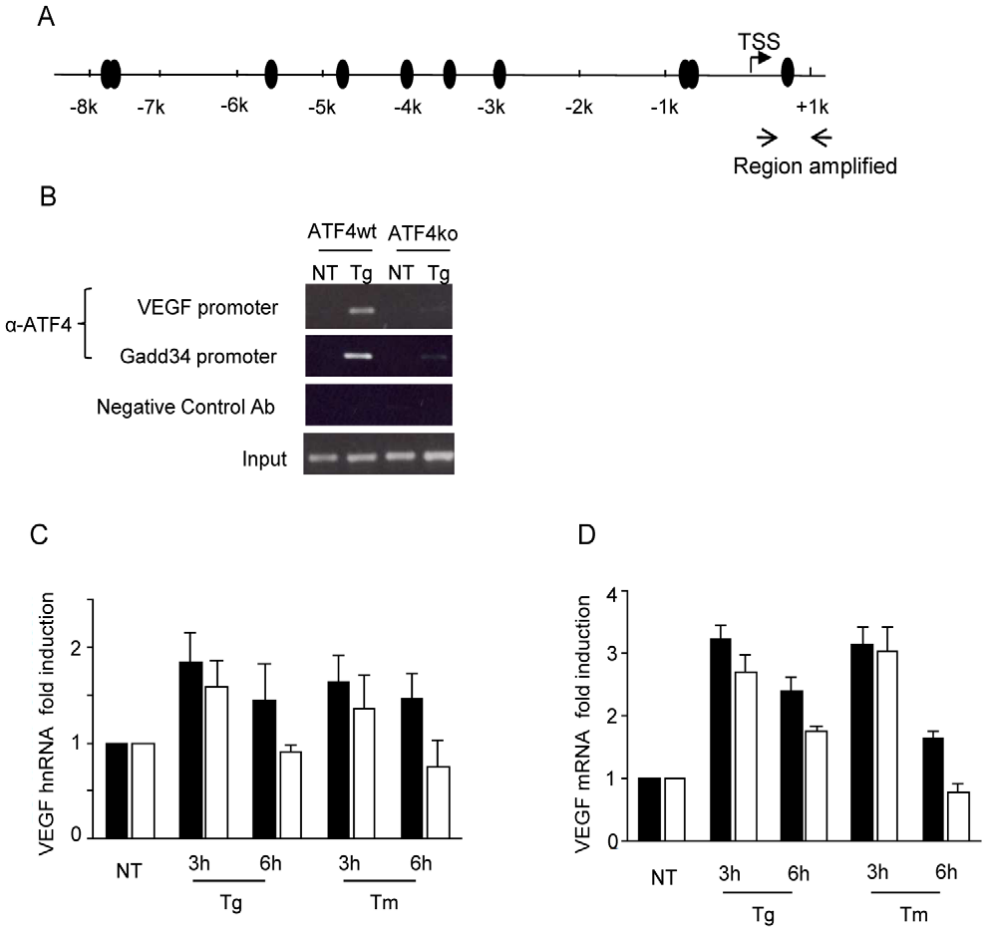
ATF4 contributes to VEGF expression and binds to the mouse promoter in a stress inducible manner. (**A**) Potential ATF4 sites in the mouse VEGF promoter (**B**) Cross-linked chromatin from ATF4 wild-type or null MEFs that were untreated (NT) or thapsigargin-treated (Tg) for 6 h was immunoprecipitated with anti-ATF4 or with a control antiserum (anti-BiP). Precipitated chromatin and input controls were used for PCR amplification. (**C–D**) ATF4 wild-type (black) or null (white) were cultured in normal media (NT), thapsigargin-treated (1 µM), or tunicamycin-treated (2.5 µg/ml) for 3 and 6 hours. Total RNA from the indicated samples was subjected to qRT-PCR, and *VEGF* hnRNA (**C**) or *VEGF* mRNA (**D**) levels were determined and represented as described above. Experiments were performed in triplicate (values are mean ± SD).

Inspection of the human, mouse and rat *VEGFA* promoters also revealed several potential ATF4 binding sites (Figure 5A and S4). To determine if any of these sites was occupied by ATF4 in response to ER stress, we performed ChIP assays in ATF4 wild-type (ATF4 wt) and null (ATF4 ko) MEFs. We were unable to detect binding of ATF4 to any of the seven potential sites upstream of the transcription start site in response to thapsigargin treatment (data not shown). However, we did detect stress-inducible binding of ATF4 to a site at position +900 relative to the transcription start site in the wild-type ATF4 MEFs but not in the ATF4 null MEFs (Figure 5B). Similar to the wild-type MEFs, we were unable to detect ATF4 binding to any of the five upstream regions in the rat promoter (Figure S6). However, unlike the human and mouse promoter, there does not appear to be a site downstream of the transcription start site in the rat promoter that corresponds to the one used in murine cells. To determine the contribution of ATF4 to *VEGFA* transcription in response to UPR stress inducers, we analyzed steady state *VEGFA* hnRNA (Figure 5C) and mRNA (Figure 5D) levels in ATF4 wild-type and null MEFs. Although the fold increase in total *VEGFA* mRNA in response to ER stress was not very dramatic in either the wild-type or null cells, it was consistently slightly higher in the ATF4 wt MEFs.

### ATF6 does not significantly contribute directly to *VEGFA* expression

**Figure 6:**
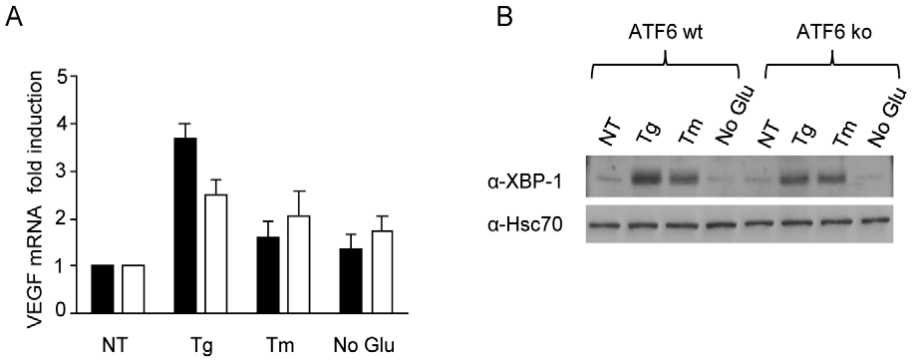
ATF6 does not significantly contribute to VEGF expression. (**A**) ATF6 wild-type (black) or null (white) MEFs were untreated (NT), thapsigargin-treated (1 µM), tunicamycin-treated (2.5 µg/ml) or treated with media lacking glucose (No Glu) for 6 hours. Total RNA from the indicated samples was subjected to qRT-PCR, and the *VEGF* mRNA levels were determined. (**B**) XBP-1(S) was detected using western blot analysis on cell lysates from ATF6 wild-type and null MEFs that were untreated or treated as indicated. Experiments were performed in triplicate (values are mean ± SD).

Although there were no obvious potential ATF6 binding sites in the human or rat *VEGFA* promoters, there was one potential ATF6 binding site in the mouse *VEGFA* gene at +1.4 kb relative to the transcription start site. Thus, we also examined the potential contribution of ATF6 in regulating *VEGFA* transcription. qRT-PCR analysis of *VEGFA* mRNA in ATF6 wild-type (ATF6 wt) and null (ATF6 ko) MEFs revealed that ATF6 does not appear to play a significant role in up-regulating *VEGFA* expression in response to either tunicamycin treatment or incubation in media lacking glucose (Figure 6A). However, in response to thapsigargin treatment there was a modest, albeit significant, increase in total *VEGFA* mRNA in ATF6 wild-type cells compared to null cells. The difference between the stresses in inducing *VEGFA* transcripts was a bit puzzling. Because XBP-1 transcription is regulated by ATF6 and our analysis revealed that XBP-1(S) binds to the *VEGFA* promoter, we examined XBP-1(S) levels in the two lines in response to the various UPR inducers. Western blot analysis revealed a more dramatic increase in XBP-1(S) protein levels in the thapsigargin-treated ATF6 wild-type cells compared to the null cells (Figure 6B), whereas there was no obvious difference between the two tunicamycin-treated cell lines. Somewhat surprisingly, media lacking glucose did not induce XBP-1(S) in either cell line. These data imply that the higher levels of *VEGFA* mRNA observed in the thapsigargin-treated wild-type cells compared to the null cells might be partly due to a more robust increase in XBP-1(S) levels in the wild-type cells.

### UPR activation increases VEGFA protein levels

**Figure 7:**
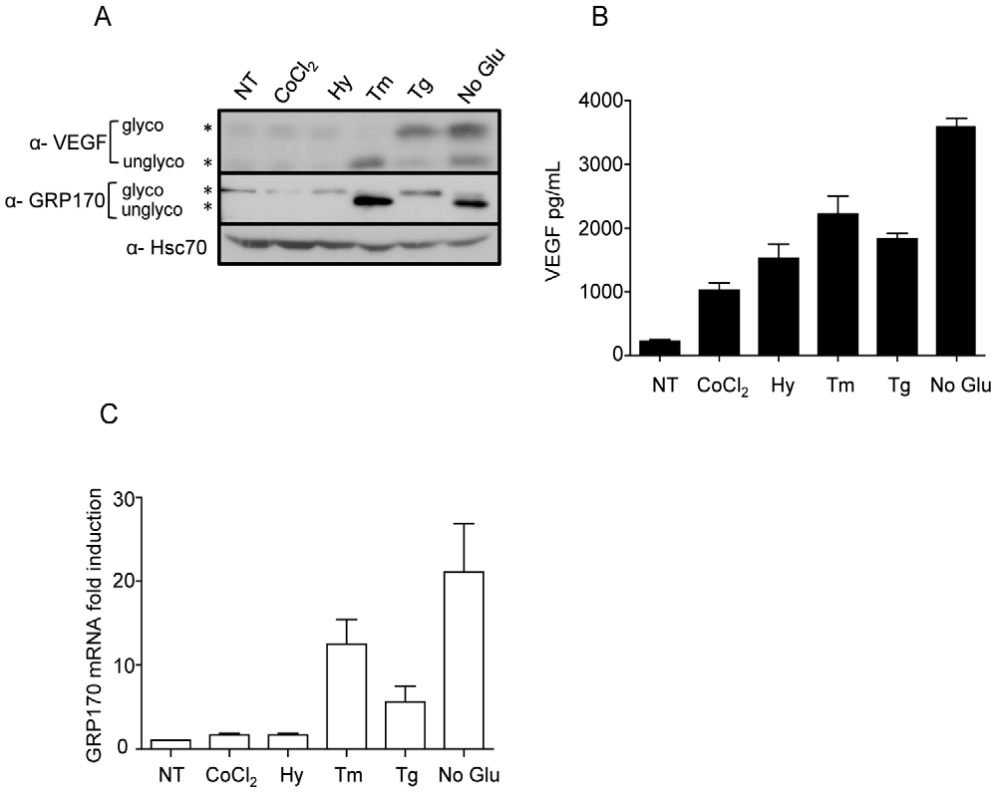
UPR activation increases VEGF protein levels and secretion. (**A**) C6 cells were left untreated or treated as indicated for 24 hours. Western blot analyses on cell lysates were performed to detect VEGF protein levels. Hsc70 was used as a loading control. (**B**) Conditioned media from untreated and treated cells was analyzed for VEGF secretion by ELISA. (**C**) Total RNA from the indicated samples was subjected to qRT-PCR, and GRP170 mRNA levels were determined and expressed relative to the control cells.

Lastly, we measured the effects of the UPR and hypoxia signaling pathways on VEGFA protein levels in the cells (Figure 7A) and in the culture supernatant (Figure 7B). We could readily detect VEGFA in lysates obtained from cells treated with all three of the UPR inducers but not in cells cultured in the hypoxia chamber or treated with CoCl_2_ (Figure 7A), in spite of the fact that hypoxia increased *VEGFA* mRNA in this cell line to about the same level as tunicamycin treatment. A faster migrating unglycosylated VEGFA was detected in cells treated with tunicamycin and no glucose media, as these stressors are known to inhibit protein glycosylation. In spite of our inability to detect VEGFA in hypoxia treated cells, when media from cells treated with the various UPR inducers and hypoxia were examined, we readily detected an increase in VEGFA secretion with all stressors (Figure 7B). However, the combination of cell-associated and secreted VEGFA with each of the treatments is not consistent with the mRNA levels. For example, although *VEGFA* mRNA levels were highest in cells treated with thapsigargin compared to any of the other four stress conditions (Figure 1A), it was not secreted as well from these cells as from tunicamycin or no glucose treated cells. Additionally, although both hypoxia and tunicamycin treatment resulted in similar increases in VEGFA transcripts, tunicamycin treatment resulted in the secretion of greater quantities of VEGFA (Figure 7B). Previous studies demonstrated that the ER stress-inducible chaperone GRP170/ORP150 plays an important role in VEGFA processing and secretion in the C6 cells [36]. Thus we examined the effects of the various stressors on GRP170 induction at both the mRNA and protein levels. We found that tunicamycin and no-glucose were potent inducers of GRP170 mRNA (Figure 7C) and protein (Figure 7A), whereas thapsigargin only modestly induced this chaperone and hypoxia had almost no effect on GRP170 levels. Thus the relatively high levels of VEGFA secretion in tunicamycin and no-glucose treated cells are consistent with the increased GRP170 levels in these cells.

## Discussion

Angiogenesis is a normal physiological process that is important for embryonic development as well as wound healing [25], [37]. It is carefully controlled by a large number of secreted factors that bind to receptors on endothelial cells, as well as negative regulators that inhibit angiogenesis through direct effects on endothelial cells or indirect effects on growth factor mobilization and activation [24], [37]. Recent studies have shown that VEGFA, one of the major proangiogenic factors, is a target of the UPR. Our microarray analysis of UPR targets in thapsigargin-treated Daoy cells confirmed *VEGFA* induction, but unexpectedly revealed that there was a significant up-regulation of 12 additional positive regulators of angiogenesis, including secreted proangiogenic factors, cytokines, and transcription factors that positively regulate proangiogenic factors, as well as a decrease in one negative regulator of angiogenesis. This argues that the regulation of angiogenesis is likely to be an important function of the UPR. Of note, the UPR was a potent inducer of IL8 expression in multiple cells lines (Figure 1D). A recent report demonstrated that addition of IL8 to endothelial cells can induce *VEGFA* mRNA and protein levels in a HIF1α-independent manner that requires NFκB activation [45]. Because ER stress also leads to NFκB activation, it is conceivable that IL8 contributes to VEGF induction during UPR activation in some of our lines. However, although the NB1691 cell line potently up-regulates IL8, it does not induce *VEGFA* mRNA levels on UPR activation. The reason for this is not known as other UPR targets are clearly activated in this line and *VEGF* is induced by hypoxia.

The major function of the UPR is thought to be restoring or maintaining ER homeostasis in response to an inadequate or toxic environment that adversely affects this organelle and its ability to fold and assemble proteins. Thus it is perhaps not surprising that one mechanism for doing this would be to increase the supply of blood flow to the affected cells so that more nutrients and oxygen can be delivered and waste and other toxic products could be taken away. In keeping with this possibility, physiological processes like wound healing require increased vascularization [46], and studies show evidence of UPR activation in the affected cells [47]. Similarly the placenta must be highly vascularized in order to supply adequate quantities of oxygen and nutrients to the developing fetus and to remove toxic waste products. Although there are not data to demonstrate that the UPR is normally activated in the developing trophoblasts, a recent report found that Ire1 was required for normal placenta vascularization and fetal development [19]. This aspect of the UPR might also be used in pathological conditions like cancer [48], [49] and ischemia [50] to stimulate angiogenesis, as in both cases there is evidence of UPR activation [2].

We focused our further analysis on VEGFA because it is the best characterized and most potent endothelial growth factor that promotes angiogenesis and is a target of cancer therapy. Recently a study has shown that *VEGFA* mRNA can be up-regulated in cultured cells by the UPR inducers, thapsigargin and tunicamycin [35]. We confirmed the role of the UPR in up-regulating *VEGFA* expression in various cell lines and also shown that the UPR is a much better inducer of *VEGFA* mRNA than hypoxia in a number of transformed and non-transformed cell lines, further arguing that this represents a normal function of the UPR. We next focused our attention on the mechanism of increased *VEGFA* mRNA expression via UPR and hypoxia signaling pathways using the C6 rat glioma cell line. The UPR increases *VEGFA* mRNA stability as well as the transcription rate of the gene to an even greater extent than that achieved with hypoxia. Several stress-activated protein kinases, including AMPK, have been reported to increase *VEGFA* mRNA stability. The activation of the AMP kinase has been linked to low glucose levels that result in diminished ATP production [51]. In addition to nutrient deprivation, other metabolic stresses such as hypoxia, oxidative stress and exercise lead to activated AMPK [52]. In DU145 prostate carcinoma cells cultured without glucose, JNK was shown to act upstream of AMPK pathway to increase *VEGFA* mRNA stability [42]. However, in our analysis of the C6 cells treatment with SP600125 the JNK inhibitor had no effect on *VEGFA* mRNA stability, whereas treatment with the AMPK inhibitor enhanced its stability in response to both thapsigargin and no glucose, suggesting that conventional UPR inducing agents can also activate AMPK.

Next, we assessed the importance of the major UPR-induced transcription factors (*i.e.,* XBP-1(S), ATF4 and ATF6) in mediating *VEGFA* transcription. Most recently, Ghosh R *et al* [35], demonstrated that Ire1 null MEFs, which cannot splice XBP-1 and induce its downstream targets, have a significant reduction in *VEGFA* mRNA expression compared to the Ire1 wild-type cells when treated with thapsigargin. Using a VEGFA-promoter-luciferase assay and chromatin immunoprecipitation they also showed that exogenously supplied XBP-1 can bind to the VEGFA promoter and up-regulate *VEGFA* mRNA expression. Our studies reveal direct binding of endogenous XBP-1 to two distinct sites in the rat *VEGFA* promoter in response to ER stress. Our qRT-PCR data showed an increase in the *VEGFA* transcription rate in the XBP-1 wild-type cells treated with various UPR inducers compared with the XBP-1 null cells. The increased levels of *VEGFA* mRNA observed in the wild-type cells was primarily due to an increase in the transcription rate of the gene, since these inducers had little effect on the stability of *VEGFA* transcript in this cell line (data not shown). Previously published data showed that tumors derived from U87 cells expressing an Ire1 dominant negative construct developed smaller tumors with decreased vascularization as compared to tumors from control cells [53]. In addition to this study, two independent reports have also demonstrated a role for XBP-1 in tumor establishment, growth and angiogenesis [54], [55]. However, the former study found that when XBP-1 deficient cells are treated with extreme hypoxia/anoxia (pO_2_<0.02%) *in vitro*, there is no defect in secretion of the proangiogenic factors, VEGFA and bFGF, as compared to wild-type cells. As these extremely low O_2_ conditions would be expected to induce both the UPR and the HIF pathways, it is conceivable that in the absence of XBP-1, HIF1α and 2α are able to compensate. In support of this possibility, there are HIF binding sites in close proximity with the XBP-1 “A” site occupied in response to ER stress (Figure 4B) in all three species. As tumor cells, ischemic tissue, and wounds are likely to activate both types of stress pathways, it will be important to understand the overlap and relative contribution of each factor in a physiological setting.

Several studies have demonstrated a role for ATF4 in mediating expression of *VEGFA* in response to various stimuli such as homocysteine [56], arsenite [57], oxidized phospholipids [58], and osteopontin [59]. Arsenite is an oxidative stressor that stimulates ATF4 binding to the *VEGFA* promoter in a human retinal pigment epithelial cell line at position +1767 relative to the transcription start site [57]. Similar observations by another group demonstrated that ATF4 binds to the same AARE site in the *VEGFA* promoter when a human umbilical vein endothelial cell line was stimulated with oxidized phospholipids [58]. These results are in accordance with data published by Ghosh et al reporting a PERK-ATF4 dependent up-regulation of *VEGFA* expression. Using both PERK and ATF4 null MEFs treated with thapsigargin, they showed that *VEGFA* mRNA levels were decreased as compared to the corresponding wild-type MEFs, and demonstrated binding of ectopically expressed ATF4 to the *VEGFA* promoter in cells treated with thapsigargin. Using ChIP assays, we confirmed that ATF4 contributes to *VEGFA* transcription and furthermore demonstrated that endogenous ATF4 binds to a region ∼+0.9 kb downstream of the transcription start site in mouse cells when treated with a UPR inducer.

Lastly, our data suggests that ATF6 does not play a significant role in directly mediating *VEGFA* mRNA expression. We observed a modest increase in *VEGFA* mRNA in the ATF6 wild-type MEFs compared to the ATF6 null cells when treated with thapsigargin but not the other stressors. This induction was most likely due to an increase in the XBP-1(S) protein levels observed only in thapsigargin-treated ATF6 wild-type cells. The VEGFA promoter-reporter assay performed by Ghosh et al, showed that over-expression of the transcription factor ATF6 increases luciferase activity ∼6 fold compared to empty vector. The reporter construct used in this assay was derived by inserting ∼1 kb of the sequence upstream of the mouse *VEGFA* transcription start site, in front of the luciferase gene. Using two different programs to identify transcription factor binding sites in this region of the mouse *VEGFA* promoter, we were unable to identify any potential ATF6 sites. However, this region contains a potential binding site for XBP-1, which this group reported could bind to ectopically expressed XBP-1 using ChIP assays. However, we were unable to detect binding of endogenous XBP-1 to this same site (data not shown).

In addition to the role of UPR-inducible transcription factors in mediating *VEGFA* expression, there are data showing that the stress inducible ER chaperone ORP150/GRP170 plays a role in post-translational processing/secretion of VEGFA [36]. Ectopic expression of ORP150 in C6 cells increased VEGFA secretion, whereas decreasing ORP150 levels with an antisense construct resulted in the retention of VEGFA in the ER. Furthermore, tumors arising from the antisense ORP150 C6 glioma transfectants demonstrate an initial phase of growth comparable to the wild-type glioma cells which was followed by marked regression and decreased angiogenesis within 8 days. Our analysis of VEGFA secretion in C6 cells revealed that although hypoxia and tunicamycin lead to similar increases in *VEGFA* mRNA, that more VEGFA was secreted from the tunicamycin treated cells, which had higher levels of ORP150/GRP170 mRNA and protein levels. This correlation is further underscored by the finding that although thapsigargin was the strongest inducer of *VEGFA* mRNA, it caused a less robust up-regulation of GRP170 and less VEGFA was secreted from thapsigargin treated cells than from either tunicamycin or no-glucose treated cells.

In conclusion, using microarray analysis we found a significant up-regulation of a large proportion of positive regulators of angiogenesis in response to ER stress and verified four of these using qRT-PCR assays. Our studies revealed that in some cell lines UPR activation enhanced *VEGFA* mRNA and protein expression more potently than hypoxia and that this was achieved through a combination of transcriptional as well as post-transcriptional mechanisms. Thus, the UPR is likely to be an important regulator of angiogenesis in normal physiological settings as well as pathological conditions like cancer and ischemia and may synergize with the well-studied HIF pathway activated by hypoxia.

## Materials and Methods

### Cell Culture and Stress Induction

Daoy human medulloblastoma cell line [60], C6 rat glioma cell line [61], XBP-1 wild-type and null MEFs, ATF6 wild-type and null MEFs, and NIH3T3 mouse fibroblasts were maintained in DMEM supplemented with 10% fetal bovine serum, 2 mM glutamine and 1% antibiotic-antimycotic at 37°C in a 5% CO_2_ incubator. NB1691 and SKNAS human neuroblastoma cell lines were cultured in RPMI 1640 supplemented with 10% fetal bovine serum and 2 mM glutamine. Primary wild-type and ATF4 null MEFs were propagated in cell culture as previously described [9]. Cells were plated and left untreated (NT) or treated with thapsigargin (Tg, 1 µM), tunicamycin (Tm, 2.5 µg/ml), homocysteine (HCys, 10 mM), CoCl_2_ (100 µM), media lacking glucose (No Glu) (DMEM cat.no.11966-GIBCO and RPMI cat. no.11879-GIBCO), or cultured in a hypoxia chamber containing 1% O_2_ for the indicated periods of time.

### mRNA and hnRNA Quantification by qRT-PCR

Total RNA was extracted using the RNeasy Qiagen mini-prep kit according to the manufacturer's protocol. RNA samples were subjected to qRT-PCR, and reactions were done in duplicate using a TaqMan One-Step PCR Master Mix kit. Amplification of the corresponding genes was achieved using specific primers and probe set and measured continuously using an ABI 7900 HTI Detection System. Where indicated, VEGF hnRNA was measured using qRT-PCR primers and probe across intron 1 and exon 2, for the rat gene and across exon 3 and intron 3, for the mouse gene. The signal obtained for measuring both, mRNA and hnRNA, was compared relative to 18S rRNA internal control. A recent study detected down-regulation of ribosomal RNA by ER stress [62]. However, in our study the 18S rRNA cycle threshold (Ct) values obtained by qRT-PCR remained relatively unchanged in the presence of ER stress arguing that 18S rRNA levels were not changing with the conditions used. Table S1 contains a list of primers and probes used in this study. The value for untreated cells was set to 1 and the value for the various treatments was presented as a fraction of this number. In the case of wild-type and null cells, the untreated value for each cell type was set to 1 unless otherwise indicated.

### mRNA Stability Assay

C6 cells were pre-incubated in normal complete media, media containing thapsigargin or no glucose, or in a hypoxia chamber for 6 hours. Actinomycin D (5 µg/ml- Sigma Aldrich) was added to each test set, and the cells were reincubated for the indicated times. Total RNA was extracted and VEGF mRNA was analyzed by qRT-PCR.

### Chromatin Immunoprecipitation

Chromatin immunoprecipitation (ChIP) analysis was performed using a ChIP kit (Upstate Biotechnology) according to the manufacturer's instructions. Briefly, cells were incubated for the indicated times with or without different stress inducers. Formaldehyde was then added (final concentration, 1%), and cells were incubated for 10 minutes at room temperature to stabilize DNA-protein interactions. Cross-linking was stopped by the addition of glycine (final concentration, 0.125 M). Cell extracts were sonicated with a Branson Sonifier 250 (VWR) for 5 bursts at 10 seconds each at 20% power output to shear DNA to 1 kb or less. Extracts from 10^7^ cells were incubated overnight with antibodies against ATF4 generously provided by Dr. David Ron (University of Cambridge, U.K.), rabbit anti-XBP-1(S) polyclonal antiserum (Santa Cruz Biotechnology sc-7160X) or rabbit anti-BiP polyclonal antiserum [63], which served as a negative control. Two percent of the extract volume was removed before immunoprecipitation and served as input control. DNA fragments from immunoprecipitated complexes and input controls were released by heating at 65°C overnight and purified using the PCR purification kit (QIAquick, Qiagen) according to the manufacturer's protocol. Purified immunoprecipitated DNA and input DNA were then analyzed by PCR. Table S2 contains a list of primers used in this study.

### Western Blotting

For Western blotting cells were lysed in Nonidet P-40 lysing buffer (50 mM Tris-HCl (pH7.5), 150 mM NaCl, 0.5% Nonidet P-40, and 0.5% deoxycholic acid) for 30 minutes on ice. The proteins were electrophoresed under reducing conditions, transferred to a PVDF membrane and probed with the indicated primary antibodies: Rabbit anti-XBP-1(S), rabbit anti-ATF4, goat anti-Hsc70, and goat anti-Lamin B1 were purchased from Santa Cruz Biotechnology, mouse anti-HIF1α and mouse anti-BNIP3 from Abcam, and rabbit anti-phosho-AMP kinase from Cell Signaling. Rabbit anti-CHOP has been described previously [8]. Isolation of nuclear and cytosolic fraction for detecting HIF1α by Western blotting was performed as previously described [64]. Blots were incubated with the appropriate HRP-conjugated secondary antibody, and proteins were visualized using the Pierce enhanced chemiluminescent substrate (Thermo Scientific).

### Microarray Gene Expression Analysis

Total RNA (5–10 µg) was processed according to the Affymetrix eukaryote one-cycle target labeling protocol (http://www.affymetrix.com/support/technical/manual/expression_manual.affx) at the Hartwell center microarray core at St Jude Children's Research Hospital. Biotin-labeled cRNA (15 µg) was hybridized overnight at 45°C to the human HG-U133 Plus 2.0 GeneChip array, which interrogates more than 54,000 human transcripts and ESTs. After staining and washing, arrays were scanned and expression values summarized using the MAS5 algorithm as implemented in the GCOS v1.4 software (Affymetrix, Santa Clara, CA). Signals were normalized for each array by scaling to a 2% trimmed mean of 500. Detection calls (Present, Absent and Marginal) were determined using the default parameters of the software. Signal values were log2-transformed prior to analysis. Differential expression between thapsigargin-treated and untreated cells was determined from two independent experiments using the Local Pooled Error t-test(1) (S-Plus 6.2, TIBCO, Palo Alto, CA). False discovery estimates were calculated as described [65]. Hierarchical clustering was performed using the Spotfire Decision Site 9.0 software (TIBCO). Probe set annotations were obtained from the Affymetrix website (http://www.affymetrix.com/analysis/index.affx). Gene ontology and network analysis was performed using Metacore from GeneGo Inc. (St. Joseph, MI). All data is MIAME compliant, and the raw data has been deposited in GEO, a MIAME compliant database, accession number: GSE21979.

### Statistical analysis

All results are expressed as mean values plus or minus SD from triplicate measurements performed in 2 to 4 independent experiments producing similar results.

## Supporting Information

Figure S1 HIF signaling pathways are not activated by UPR inducers nor are UPR targets activated by hypoxia. C6 cells were treated with 100 µM CoCl2, 1% O2 hypoxia (Hy), 2.5 µg/ml tunicamycin (Tm), 1 µM thapsigargin (Tg), or no glucose media (No Glu) for 24 hours. Western blot analysis was performed to measure (A) BNIP3 and CHOP protein levels in the cytosolic fraction. Hsc70 was used as a loading control. (B) HIF1α levels were determined in the nuclear fraction using Lamin B1 as control and CHOP levels were determined in the cytosolic fraction using Hsc70 as control.
(0.21 MB TIF)

Figure S2 UPR inducing agents up-regulate CHOP and BiP mRNA. Daoy, C6, NB1691, SKNAS and NIH3T3 cells were treated with 100 µM CoCl2, 1% O2 hypoxia (Hy), 2.5 µg/ml tunicamycin (Tm), 1 µM thapsigargin (Tg), or no glucose media (No Glu) for 24 hours. RNA was extracted for qRT-PCR analysis and expression levels of CHOP mRNA (black bars) and BiP mRNA (white bars) were determined relative to 18SrRNA. Experiments were performed in triplicate (values are mean ± SD).
(0.26 MB TIF)

Figure S3 Basal levels of proangiogenic factor expression in different cell lines. Daoy, NB1691, SKNAS, C6 and NIH3T3 cells were treated with 100 µM CoCl2, 1% O2 hypoxia (Hy), 2.5 µg/ml tunicamycin (Tm), 1 µM thapsigargin (Tg), or no glucose media (No Glu) for 24 hours. RNA was extracted for qRT-PCR analysis and basal levels of expression for (A) VEGF (B) angiogenin (C) FGF2 and (D) IL-8 were determined relative to 18SrRNA. Experiments were performed in triplicate (values are mean ± SD).
(0.21 MB TIF)

Figure S4 Potential binding sites of UPR downstream transcription factors in human, mouse and rat VEGF promoter. Two online softwares, rVista and TRANSFAC were used to screen potential binding sequences of transcription factors, XBP-1 (cyan), ATF4 (green), HIF (red) and ATF6 (yellow) in a 9 kb upstream promoter region of human, mouse and rat VEGF gene.
(0.20 MB TIF)

Figure S5 Basal levels of VEGF hnRNA in XBP-1 wild-type and null MEFs. XBP-1 wild-type MEFs (black) and null MEFs (white) were untreated (NT), Thapsigargin-treated (1 µM), treated with media lacking glucose (No Glu) or Homocysteine-treated (HCys, 10 mM) for 8 h and 14 h. Total RNA from the indicated samples was subjected to qRT-PCR and VEGF hnRNA/18S ratios were determined relative to the control untreated samples.
(0.08 MB TIF)

Figure S6 ATF4 does not appear to bind to the rat VEGF promoter. (A) Potential ATF4 sites in the rat VEGF promoter. (B) Cross-linked chromatin from C6 cells that were untreated (NT), Thapsigargin-treated (Tg), or incubated in No glucose media (No Glu) for 8 h were immunoprecipitated with anti-ATF4. As positive control, primers spanning the ATF4 binding region on the CHOP promoter were used to PCR amplify the anti-ATF4 precipitated chromatin (C) CHOP protein levels were determined using Western blot analysis in the C6 cells that were used in the ChIP assays.
(0.23 MB TIF)

Table S1 (0.03 MB DOC)

Table S2 (0.03 MB DOC)
