# Supplementary figures and images for: Transcriptional and Post-Transcriptional Regulation of Proangiogenic Factors by the Unfolded Protein Response
Source: PLoS One. 2010 Sep 2;5(9):e12521. doi: 10.1371/journal.pone.0012521 (PMC2932741; doi:10.1371/journal.pone.0012521)

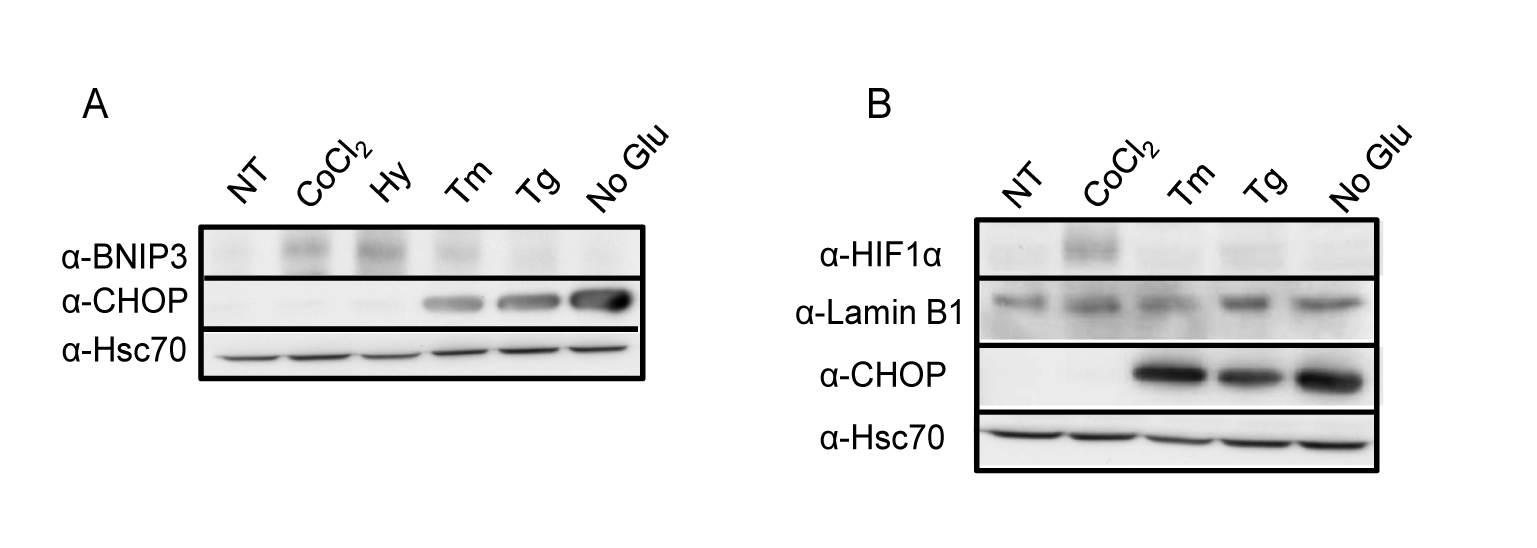

Supplement: Figure S1 — HIF signaling pathways are not activated by UPR inducers nor are UPR targets activated by hypoxia. C6 cells were treated with 100 µM CoCl2, 1% O2 hypoxia (Hy), 2.5 µg/ml tunicamycin (Tm), 1 µM thapsigargin (Tg), or no glucose media (No Glu) for 24 hours. Western blot analysis was performed to measure (A) BNIP3 and CHOP protein levels in the cytosolic fraction. Hsc70 was used as a loading control. (B) HIF1α levels were determined in the nuclear fraction using Lamin B1 as control and CHOP levels were determined in the cytosolic fraction using Hsc70 as control. (0.21 MB TIF) [file pone.0012521.s001.tif]

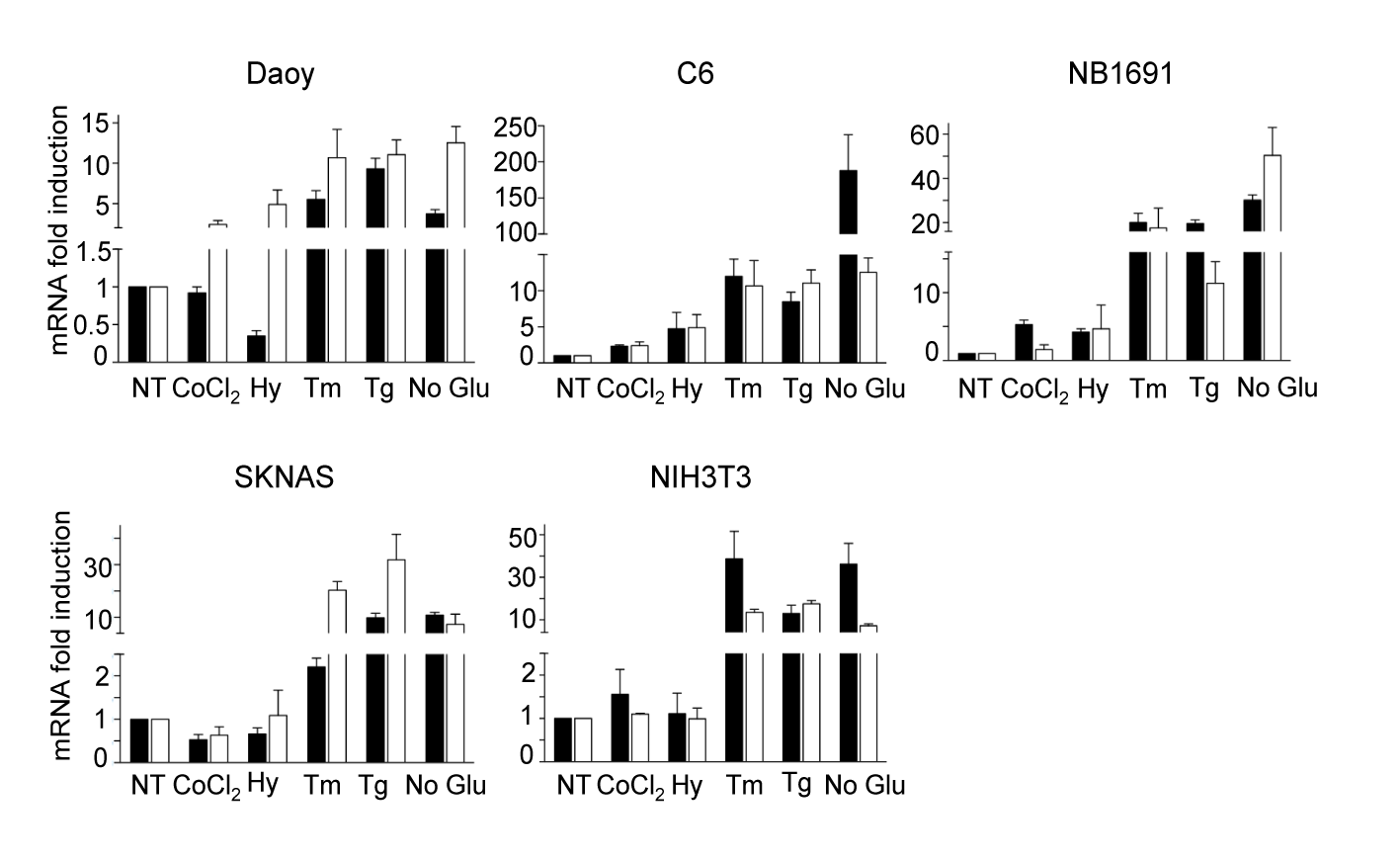

Supplement: Figure S2 — UPR inducing agents up-regulate CHOP and BiP mRNA. Daoy, C6, NB1691, SKNAS and NIH3T3 cells were treated with 100 µM CoCl2, 1% O2 hypoxia (Hy), 2.5 µg/ml tunicamycin (Tm), 1 µM thapsigargin (Tg), or no glucose media (No Glu) for 24 hours. RNA was extracted for qRT-PCR analysis and expression levels of CHOP mRNA (black bars) and BiP mRNA (white bars) were determined relative to 18SrRNA. Experiments were performed in triplicate (values are mean ± SD). (0.26 MB TIF) [file pone.0012521.s002.tif]

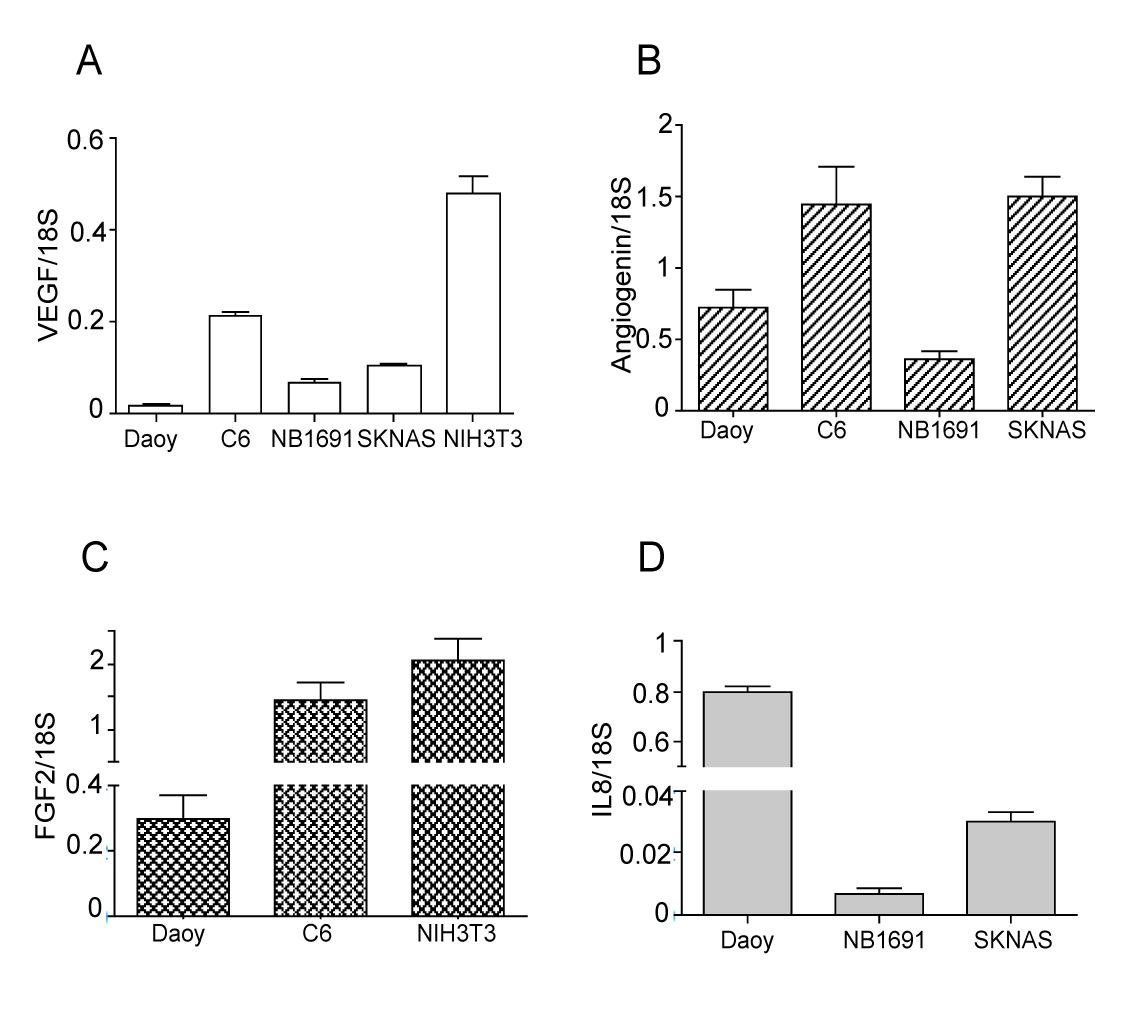

Supplement: Figure S3 — Basal levels of proangiogenic factor expression in different cell lines. Daoy, NB1691, SKNAS, C6 and NIH3T3 cells were treated with 100 µM CoCl2, 1% O2 hypoxia (Hy), 2.5 µg/ml tunicamycin (Tm), 1 µM thapsigargin (Tg), or no glucose media (No Glu) for 24 hours. RNA was extracted for qRT-PCR analysis and basal levels of expression for (A) VEGF (B) angiogenin (C) FGF2 and (D) IL-8 were determined relative to 18SrRNA. Experiments were performed in triplicate (values are mean ± SD). (0.21 MB TIF) [file pone.0012521.s003.tif]

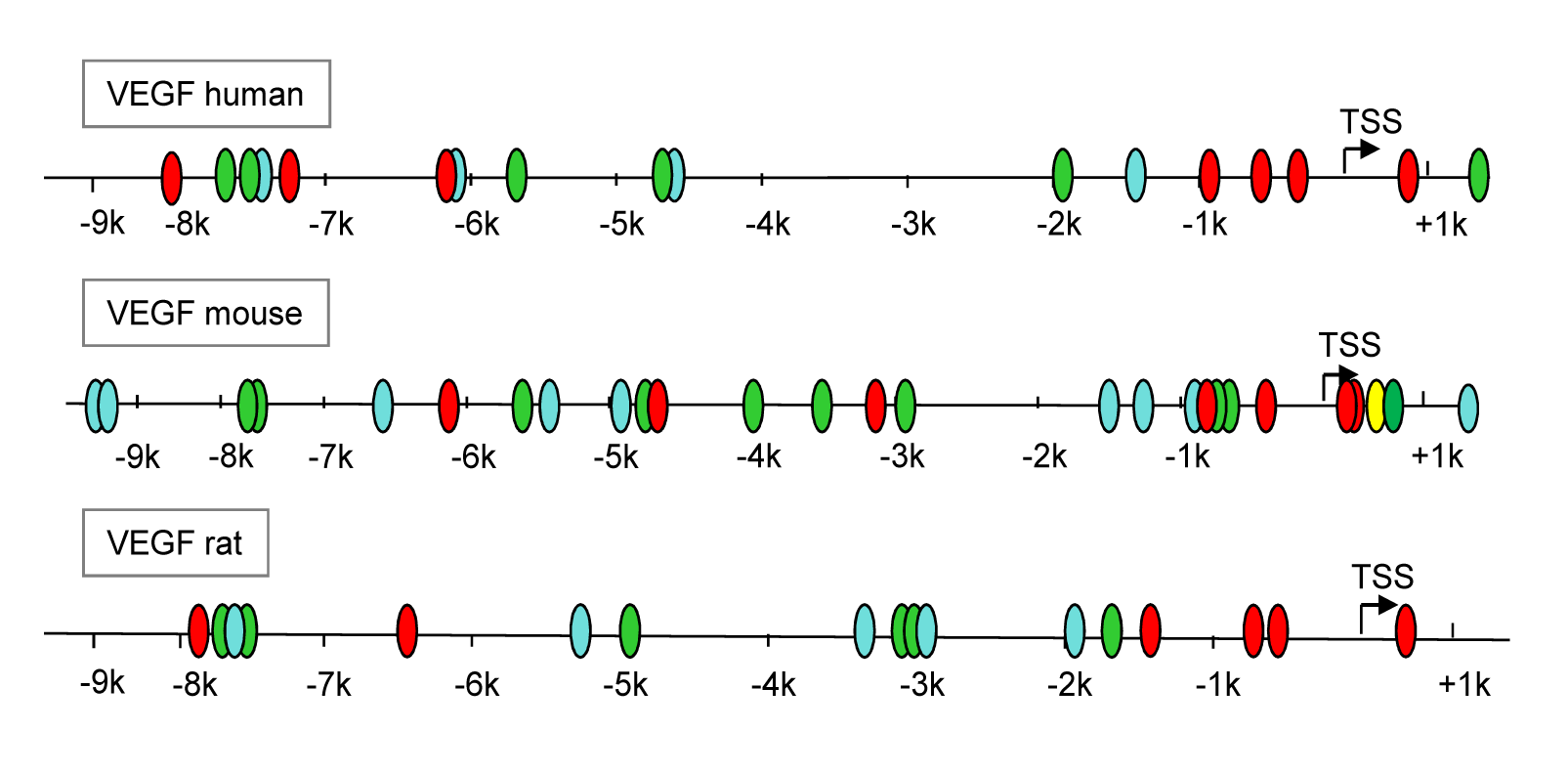

Supplement: Figure S4 — Potential binding sites of UPR downstream transcription factors in human, mouse and rat VEGF promoter. Two online softwares, rVista and TRANSFAC were used to screen potential binding sequences of transcription factors, XBP-1 (cyan), ATF4 (green), HIF (red) and ATF6 (yellow) in a 9 kb upstream promoter region of human, mouse and rat VEGF gene. (0.20 MB TIF) [file pone.0012521.s004.tif]

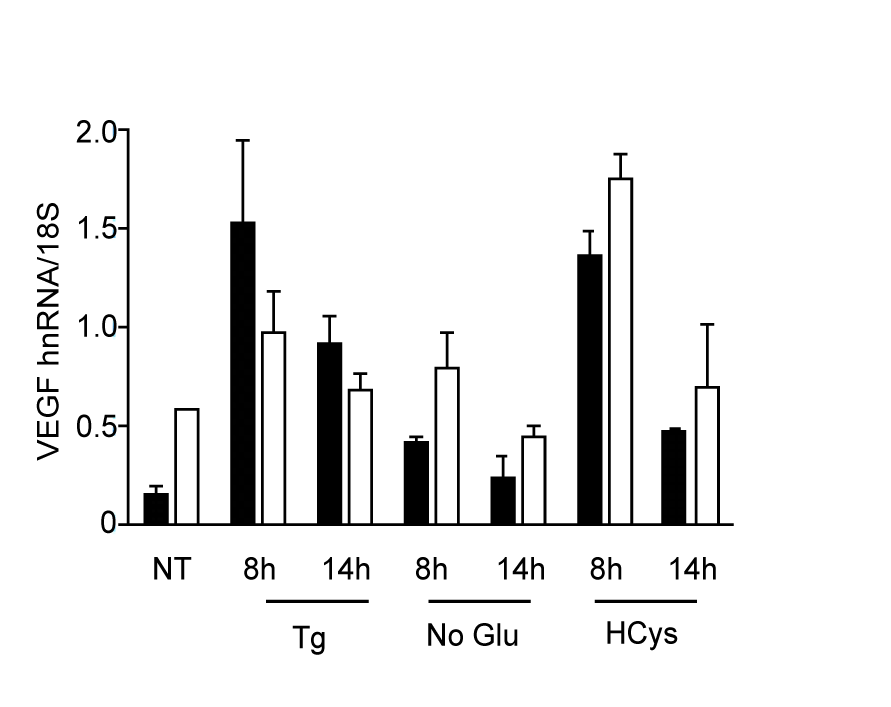

Supplement: Figure S5 — Basal levels of VEGF hnRNA in XBP-1 wild-type and null MEFs. XBP-1 wild-type MEFs (black) and null MEFs (white) were untreated (NT), Thapsigargin-treated (1 µM), treated with media lacking glucose (No Glu) or Homocysteine-treated (HCys, 10 mM) for 8 h and 14 h. Total RNA from the indicated samples was subjected to qRT-PCR and VEGF hnRNA/18S ratios were determined relative to the control untreated samples. (0.08 MB TIF) [file pone.0012521.s005.tif]

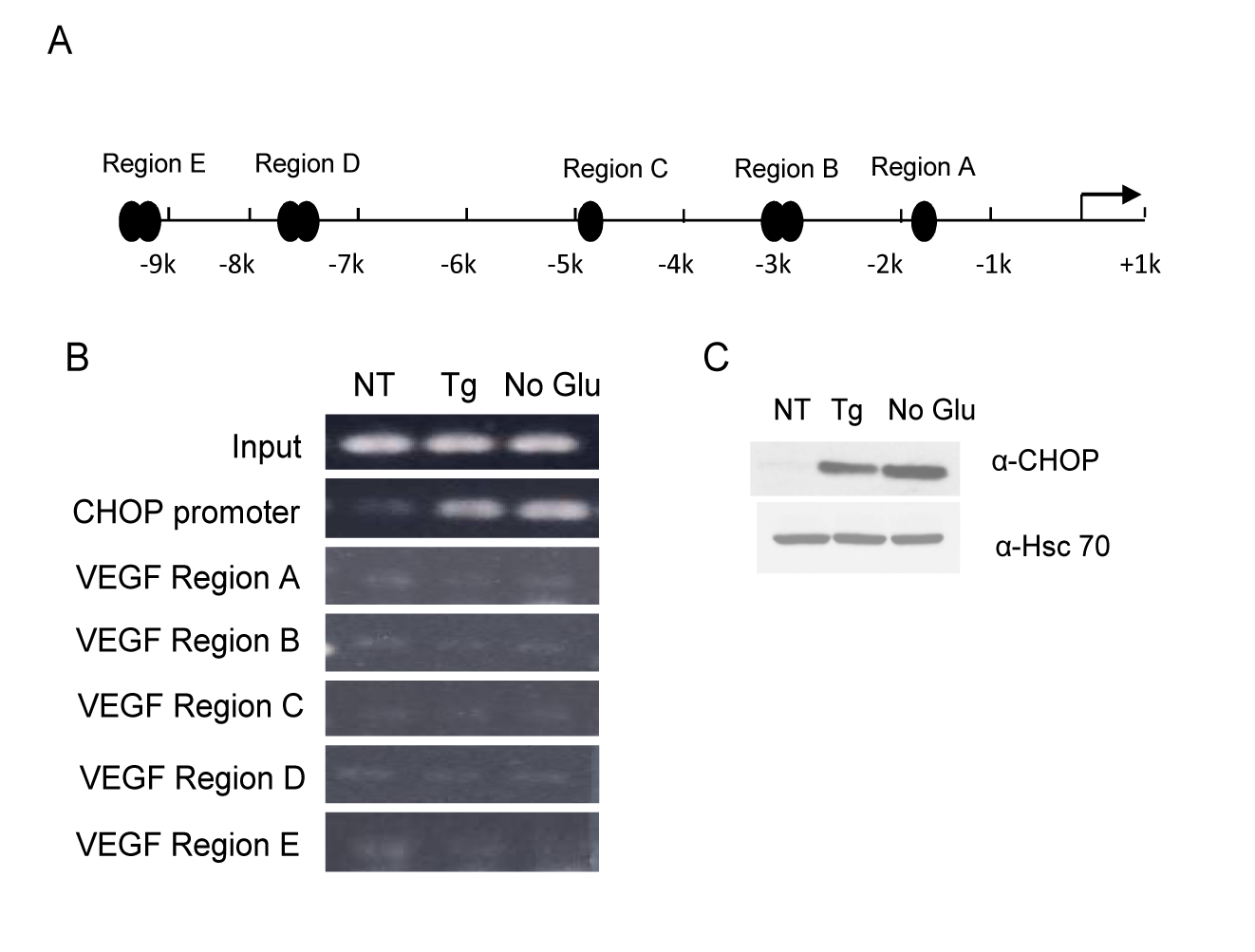

Supplement: Figure S6 — ATF4 does not appear to bind to the rat VEGF promoter. (A) Potential ATF4 sites in the rat VEGF promoter. (B) Cross-linked chromatin from C6 cells that were untreated (NT), Thapsigargin-treated (Tg), or incubated in No glucose media (No Glu) for 8 h were immunoprecipitated with anti-ATF4. As positive control, primers spanning the ATF4 binding region on the CHOP promoter were used to PCR amplify the anti-ATF4 precipitated chromatin (C) CHOP protein levels were determined using Western blot analysis in the C6 cells that were used in the ChIP assays. (0.23 MB TIF) [file pone.0012521.s006.tif]
