# Supplementary material for: Transcriptional and Post-Transcriptional Regulation of Proangiogenic Factors by the Unfolded Protein Response
Source: PLoS One. 2010 Sep 2;5(9):e12521. doi: 10.1371/journal.pone.0012521 (PMC2932741; doi:10.1371/journal.pone.0012521)
Supplement: Table S1 — (0.03 MB DOC) [file pone.0012521.s007.doc]

| **Common Name** | **Species** | **Primer** | **Probe** |
| --- | --- | --- | --- |
| VEGF(mRNA) | Human | Forward: CCTCCGAAACCATGAACTTT  Reverse: CCACTTCGTGATGATTCTGC | CCAAGTGGTCCCAGGCTGCA |
| VEGF (mRNA) | Rat/Mouse | Forward: CACTGGACCCTGGCTTTACT  Reverse: CTTCTGTCGTGGGTGCAG | CCATGCCAAGTGGTCCCAGG |
| VEGF (hnRNA) | Rat | Forward: AAGGTGAGTCCTCATGCTTGT  Reverse: GTGCAATCTCGACCCTCATA | TGGGTCCCTGTTGTCCCATTCC |
| VEGF (hnRNA) | Mouse | Forward: TCTCCTATGTGCTGGCTTTG  Reverse: CTGGGTCACTAACCACTGTGA | CCCTCCCTCTACAGATCATGCGG |
| BiP | Mouse | Forward: AGGAGACTGCTGAGGCGTAT  Reverse: GCTGGGCATCATTGAAGTAA | CTGCATGGGTAACCTTCTTTCCCAA |
| Gadd34 | Mouse | Forward: AGAGAAGAGGGAGTGGCTGA  Reverse: TTCATTCTCAGCTGGACCAC | CCCGCTTATCCCACATCACAGCT |
| GRP170 | Rat | Forward: GACAGGGTGGAGTCCGTATT  Reverse: CAAACAGGCTGGATATGGTG | TCTGGGCTATCCTCCACCAGGG |
| IL8 | Human | Forward: AGGACAAGAGCCAGGAAGAA  Reverse: ACTGCACCTTCACACAGAGC | ACTTCCAAGCTGGCCGTGGC |
| FGF2 | Human | Forward: TGGCTATGAAGGAAGATGGA  Reverse: ACTGCCCAGTTCGTTTCAGT | CCAACTGGTGTATTTCCTTGACCGG |
| Angiogenin | Human | Forward: AGGAGCCTGTGTTGGAAGAG  Reverse: AGCACGAAGACCAACAACAA | AACGCCCAGGCCCATCACC |

**Table S1. List of qRT-PCR primers and probes**
