# Supplementary material for: Transcriptional and Post-Transcriptional Regulation of Proangiogenic Factors by the Unfolded Protein Response
Source: PLoS One. 2010 Sep 2;5(9):e12521. doi: 10.1371/journal.pone.0012521 (PMC2932741; doi:10.1371/journal.pone.0012521)
Supplement: Table S2 — (0.03 MB DOC) [file pone.0012521.s008.doc]

| **Gene** | **Antibody used for ChIP** | **Species** | **Primer** |
| --- | --- | --- | --- |
| VEGF promoter- Region A | XBP-1 | Rat | Forward: GCTTCTGTCTGCCCAGCTGTCTCTC  Reverse: CAGAGAGGCTTCTCTGGAGAGGATATG |
| VEGF promoter- Region B | XBP-1 | Rat | Forward: GCTGAGAACCACTGCTGTAGAAGGTAG  Reverse: ATAATCTATGCAGCTAAGAGGCACTG |
| VEGF promoter- Region C | XBP-1 | Rat | Forward: CTTCCAGAGCAGTAAGGACACAGGC  Reverse: CTGAGAAGGCTGCATGCTGTGTGAC |
| VEGF promoter- Region D | XBP-1 | Rat | Forward: AAGTGAGAGTCCTAGCCTGGTCCT  Reverse: GGCATGTCGGCTGATGTTCGC |
| ERdj3 promoter (control) | XBP-1 | Rat | Forward: TCCTCTCTCCGGTTCTAAGGCAC  Reverse: TGGCCGCGGAGACAGGTAGT |
| VEGF promoter | ATF4 | Mouse | Forward: GCCGATTACATCAGCCCG  Reverse: CGGGTAGAAGTTGGGAACG |
| Gadd34 promoter (control) | ATF4 | Mouse | Forward: GCTCGGAAATTACGTGAGATCG  Reverse: GCGCCAACATCGTCCACGCG |
| VEGF promoter- Region A | ATF4 | Rat | Forward: TGACTGAAGAGTAGAGC  Reverse: TAATCTATGCAGCTAA |
| VEGF promoter- Region B | ATF4 | Rat | Forward: ATGAACCTCCTGAGTGTTA  Reverse: GATGGGGATTTCACCTGTC |
| VEGF promoter- Region C | ATF4 | Rat | Forward: GCTAAAGCAGAAACCAAGC  Reverse: ATGAGGCCCTGTCATTAA |
| VEGF promoter- Region D | ATF4 | Rat | Forward: CTAGTCCCCAGGGTTCTTAA  Reverse: ACAACATGGCCCCATAG |
| VEGF promoter- Region E | ATF4 | Rat | Forward: GCTTTGCTGGTTGGTAT  Reverse: GCCTTGTACTCTATGAATG |
| CHOP promoter (control) | ATF4 | Rat | Forward: GCGGACACCGGTTGCCAAACA  Reverse: CTCTGGCTCGAGAGTCTACGTTA |

**Table S2. List of primers used in the chromatin immunoprecipitation assays**
